# Multispectral Optical Remote Sensing for Water-Leak Detection

**DOI:** 10.3390/s22031057

**Published:** 2022-01-29

**Authors:** Jean-Claude Krapez, Javier Sanchis Muñoz, Christophe Mazel, Christian Chatelard, Philippe Déliot, Yves-Michel Frédéric, Philippe Barillot, Franck Hélias, Juan Barba Polo, Vincent Olichon, Guillaume Serra, Céline Brignolles, Alexandra Carvalho, Duarte Carreira, Anabela Oliveira, Elsa Alves, André B. Fortunato, Alberto Azevedo, Paolo Benetazzo, Alessandro Bertoni, Isabelle Le Goff

**Affiliations:** 1 ONERA-The French Aerospace Lab, 13300 Salon de Provence, France; christian.chatelard@onera.fr (C.C.); yves-michel.frederic@onera.fr (Y.-M.F.); philippe.barillot@onera.fr (P.B.); franck.helias@onera.fr (F.H.); 2 Galileo Geosystems, Manises, 46940 Valencia, Spain; javio.sanchis@uv.es (J.S.M.); juan@galileogeosystems.com (J.B.P.); 3 AM, Air Marine, 33850 Leognan, France; christophe.mazel@air-marine.fr (C.M.); vincent.olichon@air-marine.fr (V.O.); 4 ONERA-The French Aerospace Lab, 33000 Toulouse, France; philippe.deliot@onera.fr; 5 Water Service Department, Société du Canal de Provence, SCP, Le Tholonet, 13182 Aix-en-Provence, France; guillaume.serra@canal-de-provence.com (G.S.); celine.brignolles@canal-de-provence.com (C.B.); 6 EDIA, S.A., 7800-502 Beja, Portugal; acarvalho@edia.pt (A.C.); dcarreira@edia.pt (D.C.); 7 Hydraulics and Environment Department, National Laboratory for Civil Engineering, LNEC, 1700-066 Lisbon, Portugal; aoliveira@lnec.pt (A.O.); ealves@lnec.pt (E.A.); afortunato@lnec.pt (A.B.F.); aazevedo@lnec.pt (A.A.); 8 SGI, Studio Galli Ingegneria, 35030 Padova, Italy; paolo.benetazzo@sgi-spa.it (P.B.); alessandro.bertoni@sgi-spa.it (A.B.); 9 Formerly at Société du Canal de Provence, SCP, 13182 Aix-en-Provence, France; legoff.ochier@free.fr

**Keywords:** remote sensing, water leak, infrared, Triangle Method, Trapezoid Method, soil moisture, evaporation, thermal

## Abstract

Water losses from water distribution means have a high environmental impact in terms of natural resource depletion (water, energy, ecosystems). This work aims to develop an optical airborne surveillance service for the detection of water leaks (WADI—Water-tightness Airborne Detection Implementation) to provide water utilities with adequate and timely information on leaks in water transportation mains outside urban areas. Firstly, a series of measurement campaigns were performed with two hyperspectral cameras and a thermal infrared camera in order to select the most appropriate wavelengths and combinations thereof for best revealing high moisture areas, which are taken as a proxy for water leakage. The Temperature-Vegetation-Index method (T-VI, also known as Triangle/Trapezoid method) was found to provide the highest contrast-to-noise ratio. This preliminary work helped select the most appropriate onboard instrumentation for two types of aerial platforms, manned (MAV) and unmanned (UAV). Afterwards, a series of measurement campaigns were performed from 2017 to 2019 in an operational environment over two water distribution networks in France and Portugal. Artificial leaks were introduced and both remote sensing platforms successfully detected them when excluding the unfavorable situations of a recent rain event or high vegetation presence. With the most recent equipment configuration, known and unknown real leaks in the overflown part of a water transportation network in Portugal have been detected. A significant number of false alarms were also observed which were due either to natural water flows (groundwater exfiltration, irrigation runoff and ponds) or to vegetation-cover variability nearby water-distribution nodes. Close interaction with the water utilities, and ancillary information like topographic factors (e.g., slope orientation), are expected to reduce the false alarm rates and improve WADI’s methodology performance.

## 1. Introduction

Innovation is key for coping with future water challenges in fields like water management, land use and agriculture since water has become increasingly scarce and less predictable [1]. In addition, the balance between competing demands for water (as fostered by a growing population and increasing agricultural production) and its limited supply is believed to be threatened with speculation since the recent creation of the world’s first futures market in water [2]. Improved leak management could boost the network efficiency and optimize water source uses leading to significant environmental benefits. In Europe for example, within Member States, leakage rates vary from 7% to 50% or more [3], 60% of European cities over-exploit their groundwater resources and 50% of wetlands are endangered [4], which could get worse if a more efficient and sustainable use of water resources is not achieved.

Despite significant improvements over the past 20 years in understanding the economic management of real losses on water distribution systems, little information is available on the actual extent of transmission mains leakage, and how best to quantify and manage it. Detection of water leaks in transmission systems for water supply, irrigation, and hydropower plays a key role in water management efforts to mitigate natural resource depletion, decrease the related energy consumption and help utilities to provide cost-effective services. At present, leaks from seals on transmission mains can go undetected for years causing a significant loss of water. While leaks on large-diameter pipelines are estimated to make up less than 5% of the total number of leaks, they can account for more than 50% of the total leak-water lost. Detection of leakages in large diameter underground mains is thus a key point but its timely detection faces major challenges.

Water leakage detection has been carried out by several ground techniques that typically involve measurement of pressure differences between two valves, acoustic sounding, ground-penetrating radar or gas injection [5]. However, the use of ground methods is difficult and often inadequate for leak detection in water transmission mains [6]. Transmission main leaks are difficult to locate, generally because this type of water transportation infrastructure has one or more of the following characteristics: low pressure; low noise frequency; large diameter; non-metallic material; and few contact points for acoustic monitoring. In addition, transmission mains are often located outside urban areas in difficult-to-access remote areas. Finally, the cost of detection, location, and repair of unreported bursts on transmission mains is significantly greater on a per-km basis than on distribution mains, often by an order of magnitude or more, which can lead utilities to simply exclude transmission mains from leak detection programs. In addition to underground transportation pipelines, the problem of water leaks also affects open canals resulting in seepage to the embankments.

Therefore, there is a strong need for developing transmission mains surveillance methods that will dramatically, but also cost-effectively, speed up pipeline and canal monitoring. The technology and service described in this paper address all these expectations, taking advantage of existing remote sensing technologies.

Our methodology relies on several leak characteristics. In the close vicinity of a leak, soil moisture increases until it reaches the field capacity or possibly saturation. Thereafter, water moves through the soil pores in all directions, in particular up to the surface. Additionally, the water supplied by the leak in the root zone will benefit the vegetation already present or induce the growth of opportunistic plants nearby. Thus, underground leaks and canal seepage are expected to enhance water presence in the vicinity of the leak up to the top surface of the soil/vegetation complex.

Remote sensing techniques that are able to detect changes in water content in soil or vegetation are thus, good candidates for leak detection. They provide better temporal and spatial coverages than ground detection methods.

Airborne active/passive microwave sensors are suitable for mapping soil moisture since the received signal is sensitive to the dielectric constant of soil, hence to the moisture content. However, the depth-penetration capability depends on the sensor wavelength. In addition, the correlations between radiation and soil moisture are degraded by surface roughness [7] and variability in vegetation cover [8].

Optical methods could be an alternative, as optical properties change with moisture content in soil and vegetation. A well-known observation is the darkening of the soil surface when water is added, which results from a decrease in its reflectance in the visible spectrum. The estimation of the soil moisture content from multispectral or hyperspectral remote sensing data strongly depends on the availability of a database that is representative of all the soils encountered in the scanned area since the correlation between the moisture content and the reflectance spectrum is conditioned by, among other properties, the texture of the soil, and the presence of organic matter and crust [9]. In addition, these databases refer to bare soils, which are not the most frequent type of surfaces met in areas where leaks in water transportation means are sought. Notice that the penetration depth of optical sensors in the visible to short-wave range (i.e., [0.4–2.5 µm]) is significantly lower than for microwave sensors: the moisture content evaluated by the former type of sensor is that of the uppermost layer of the soil column.

Finally, thermal infrared (TIR) remote sensing has long been recognized as a valuable tool for evaluating soil moisture [10]. The sensed surface temperature is a strong indicator of the presence of water both in soil and in plants, which can help for crop-water stress evaluation [11]. Temperature is a state variable that evolves during the day as a function of the surface radiative fluxes (both from solar radiation and from atmospheric and environmental infrared radiation), the atmospheric sensible and latent turbulent fluxes, and the heat diffusion inside the soil. A coupling also takes place with the water transport inside soil and plants up to the atmosphere by involving phase changes into or from water-vapor through evaporation, transpiration and condensation. The main governing parameters are the surface albedo, the surface emissivity, the resistance to evapotranspiration and soil thermal inertia (or thermal effusivity). They depend on the water content at the soil surface and in the upper soil layers, and, if vegetation is present, on its water content and possible water stress (which induce stomatal closure, hence limiting water losses). Despite some antagonistic effects, the consequence of the relative contributions of all these parameters is that higher water content reduces the day–night temperature amplitude [12]. During a sunny day, moist sites show a lower temperature as compared to drier sites [13,14,15]. After sunset and before dawn, depending on the moisture levels in the sites, the temperature gap is reduced or maybe reversed [13,14,15]. Based on this interpretation several remote sensing experiments involving TIR sensors were conducted for assessing the moisture state at the Earth’s surface or the evaporation rate over vegetated lands, in particular water stress, see [10,11].

Airborne TIR has been used for water leaks and seepage detection along aqueducts, canals, and dikes for nearly forty years [15,16,17,18,19,20,21,22,23]. TIR was sometimes used independently by implementing infrared cameras or line scanners [15,17,21]. Out of the 39 sites detected as potential canal leakage sites in [15], 12 were verified through field analysis as actual leakage sites, giving the technique a 31% detection accuracy rate with 69% false positives. In addition, during the field checking process, no other leakage sites were discovered (no false negatives). It was concluded in [15] that, although the detection accuracy was low, the amount of time saved by checking this limited number of sites rather than the entire canal system for leakage was tremendous. Misinterpretations were commonly caused by dense natural vegetation, farm canals or drainage ditches adjacent to the main canal, smallholding ponds or low-depression areas of natural drainage. It was suggested in [15] to use color photography to supplement thermal imagery in order to help image interpretation and reduce misinterpretation errors. More recent works often combine TIR with visible imagery, but also with images obtained in other spectral bands. TIR was used simultaneously with visible and NIR (near-infrared) cameras [20] with visible, multispectral and hyperspectral cameras [16], or through a multispectral sensor spanning from the visible to the thermal infrared range [18,23]. Aerial remote sensing data have also been interpreted through a combined analysis with satellite imagery like Landsat TM [19] or Google Earth [23]. In [16,18,19,20,23], the additional visible-to-SWIR spectral bands were used as standalone information or were fused to provide various indicators like well-known vegetation indices or moisture indices (NDVI, MSAVI, CRI, SRWI, WBI, NIRRR, etc.). This additional information allowed for the increasing of TIR’s performance by reducing the number of false detections, for example by assisting in identifying sites that were likely to be confounded with seepage in the thermal imagery (e.g., dense vegetation) [20]. By simply processing a vegetation index, (namely NDVI), the NIR and red images could differentiate the tree and shadow from nearby seepage areas whereas the thermal image could not [20]. A 93% success rate was achieved for leak detection in canal systems based on a combined TIR and NDVI image analysis [20]. Even simple images from Google Earth allow rectifying the false identification of water activity provided by tree shades in the TIR images [23]. Moreover, Landsat TM data provided information on interactions of land use and vegetation with seepage zones (in that instance, using the MSAVI index proved to be more efficient than NDVI in case of low vegetation cover) [19].

Soil water content and crop water deficit have been evaluated with the so-called Triangle Method, which uses more synergistically TIR data and visible-to-NIR data [24,25,26,27,28]. The principle of this method lies in the following observation. Since the relationship between temperature and water content is different between vegetation and bare soil, the mean temperature sensed by the remote TIR sensor depends not only on the water content but also on the vegetation cover-fraction in the considered pixel. For this reason, there is an intrinsic ambiguity in the deduction of moisture, or in the simple detection of leakage spots from the mean temperature as provided by a thermal sensor integrating the infrared radiance in mixed pixels. Independent information about the vegetation cover-fraction is therefore required to eliminate the ambiguity. Introducing a vegetation index (VI) as a proxy of the vegetation cover-fraction solves the problem. Classical vegetation indexes are inferred from the visible and near-infrared (NIR) signals provided by one or two additional cameras. The temperature and the VI obtained over an area presenting a broad diversity both in cover-fraction and in humidity provide a 2D scatterplot with a roughly triangular shape, which explains the name of the method: the Triangle Method [24,25,26]. Actually, the vertex of the triangle related to dense vegetation is often truncated, giving a trapezoidal shape, see [24,25,26,27,28]. The low-temperature boundary of the scatterplot is related to pixels/surfaces with the highest moisture and evaporation/transpiration rate (“wet” edge), whereas the high-temperature boundary is related to pixels/surfaces with the lowest moisture and evaporation/transpiration rate (“dry” edge). As a consequence, a water index (or the complementary dryness index) is assigned to each point from its relative position with respect to the “wet” and “dry” edges of the triangle/trapezoid scatterplot.

More sophisticated approaches use an SVAT model (Soil Vegetation Atmosphere Transfer) to define the water content from the position of the representative point (in temperature and VI) inside the triangular scatter [25,27,28,29]. More recent works consider additional information such as the albedo [30,31,32] or the CAI—Cellulose Absorption Index [31,32] which intends to discriminate between green and senescent vegetation. Adding a third observation parameter to the classical version of the Triangle Method is expected to allow discrimination between areas that have different moisture contents but with similar coupled temperature and vegetation index values. For the crop fields analyzed in [31], the addition of the CAI proved to be more efficient than the addition of the albedo.

The Triangle/Trapezoid Method has been most often applied to satellite imagery, hence providing soil moisture maps at low resolution (60 m–1 km), see [26,28,30]. Applications to airborne remote sensing data for getting information on moisture at a higher resolution (1–30 m with a plane and in the range of 10 cm or less with a UAV) are less common [25,27,28,29,31,32,33,34,35,36]. They actually reveal higher difficulties, including the surface heterogeneity being exacerbated by the high-resolution sensors, which as a consequence, imposes a higher demand on the accuracy of the image registration process.

Since heat transfer between atmosphere, vegetation and soil is a dynamic process, the temperature signatures of dry and wet surfaces evolve with time. As a consequence, the success of TIR leak detection depends on the time chosen in the year, on the period of the day, on the delay since the last precipitations [22], and more generally, on current and past environmental conditions. An optimum time is hard to determine, especially since the vegetation has a high impact (vegetation type and height, cover-fraction, water stress, etc.). For this reason, the post-leaf season (from late autumn to early spring) is sometimes recommended to obtain a relatively unobstructed view of moist and wet areas, depending on the vegetation type and height [17]. Other investigators preferred to apply TIR in summer to maximize the chance of seeing a dry period and thereby increase the contrast between leak areas and their surroundings [18].

The optimal time during the day for a TIR investigation is still an open issue. According to Jensen [14], thermal crossovers between water and other surfaces occur at sunset and one or two hours after sunrise. The two extrema of the temperature difference between water and most other objects are then observed just before sunrise and around the mid-point between solar noon and local sunset. This would favor performing thermal measurements in the period from solar noon until sunset (day-time option) or during the hours preceding sunrise (night-time option), as inferred in [16]. Conducting the TIR measurements after sundown or before sun-up prevents solar perturbations but its success depends on the material of the water main [17,18,19]. A higher temperature over leaks during the night, as expected due to the higher thermal inertia, was not systematically observed [18]. Recent measurements performed during a whole day at several locations with wet and dry soils in a dike excavated area highlighted the fact that the contrast is at a maximum in the afternoon (the exact optimal time actually depends on the considered spot pair) and that it nearly vanishes after the sunset and before the sunrise, precluding the night-time option [16].

The H2020 WADI project (Water-tightness Airborne Detection Implementation [37]) aimed at developing an airborne water-leak detection surveillance service to provide water utilities with adequate information on leaks in water infrastructures outside urban areas, thus enabling prompt and cost-effective repairs and reducing losses in the water distribution systems. An airborne system has been chosen instead of a satellite-based one to obtain a finer resolution, ideally in the range of 0.5 m. WADI’s innovative concept consists of coupling and optimizing off-the-shelf optical remote sensing devices (multispectral and infrared cameras) and applying them on two complementary aerial platforms (manned and unmanned). The project also aimed to test and validate the concept in two real, contrasting leak detection sites. The goals of this paper are to present the WADI methodology and its improvement pathway, and showcase its performance in two contrasting sites: Provence area, France, occasionally after rainy conditions, and Alqueva, Portugal, under dry conditions. The range of tests comprised also a wide range of other environmental characteristics including soil and vegetation type. Preliminary achievements are described in [38,39,40].

This paper is organized as follows. Section 2 describes the design of the optical sensor systems after testing various spectral combinations. Section 3 presents the validation results using the selected multispectral method and instruments during a series of campaigns at different scales using both a manned aircraft platform and a UAV over several parts of the water networks of SCP (Société du Canal de Provence) in France and EDIA (Empresa de Desenvolvimento e Infra-estruturas do Alqueva), in Portugal. A detailed performance analysis of all flights is presented in Section 4, along with a discussion on the WADI methodology’s success. Finally, the conclusions are given in Section 5.

## 2. Preliminary Analysis: Selection of Optical Spectral Bands Using Hyperspectral Cameras and a Thermal Infrared Camera

### 2.1. Materials and Methods

**Figure 1:**
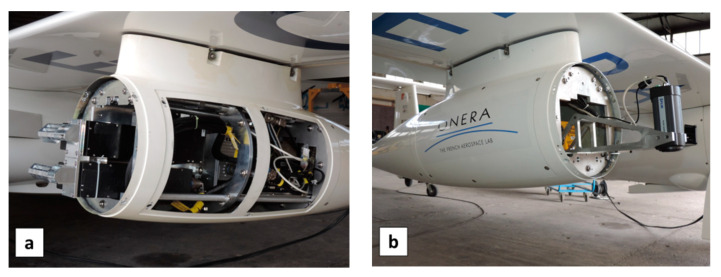
(**a**) VNIR1600 and SWIR320m-e HySpex hyperspectral cameras on-board left pod of motor-glider STEMME; (**b**) IR microbolometer camera (FLIR A325) onboard right pod.

The optical sensor systems aimed at being used onboard a plane and UAV were designed by first performing a series of airborne measurements involving a thermal infrared camera with microbolometers (first A325 and then A655sc, FLIR, Portland, OR, USA) and two hyperspectral cameras Hyspex (NEO, Oslo, Norway), one in the visible to near-infrared spectrum (VNIR: 0.4–1 µm), and another one in the shortwave infrared spectrum (SWIR: 1–2.5 µm) (Figure 1). This approach helped select the best spectral combination by comparing the responses at different wavelengths, more precisely, by comparing the values of several spectral indexes expected to differentiate between dry and humid areas. As a consequence, this helped select simpler and cheaper cameras for operational applications.

The image processing (orthorectification, atmosphere correction, and co-registration by the GeFolki software [41]) is described in [38].

The availability of continuous spectral data in the 0.4–2.5 µm range and thermal infrared data made it possible to test the effectiveness of different water indexes.

The PWI (Plant Water Index, which is also called the WBI—Water Band Index) was proposed to map vegetative water content [42]:

(1) PWI=WBI=R900/R970

where RX means reflectance measured at X nm. However, it is sensitive not only to water content but also to the vegetation–canopy structure and viewing geometry [43]. Two indexes take advantage of the liquid water absorption band centered at 1240 nm: the Normalized Difference Water Index $\mathrm{NDWI}{\;}_{1240}$ [43,44]:

(2) $$\mathrm{NDWI}{\;}_{1240}\;=\left(R858-R1240\right)/\left(R858+R1240\right)$$

and the Simple Ratio Water Index (SRWI) [43,45]:

(3) SRWI=R858/R1240

Water absorption dominated bands at 1640 and 2130 nm were used to build two Normalized Difference Water Indexes $\mathrm{NDWI}{\;}_{1640}$ and $\mathrm{NDWI}{\;}_{2130}$ [46].

(4) $${\mathrm{NDWI}}_{\;1640}=\left(R858-R1640\right)/\left(R858+R1640\right)$$

(5) $${\mathrm{NDWI}}_{\;2130}=\left(R858-R2130\right)/\left(R858+R2130\right)$$

In an attempt to estimate the vegetation water content in corn fields from an operational satellite, the bands at 1640 and 2130 nm showed better results than the NIR band at 1240 nm [46].

The following four indexes were specifically designed for evaluating the soil moisture content (SMC) in bare soils: WISOIL (Water Index SOIL [47,48]), NSMI (Normalized Soil Moisture Index [49]), NINSOL (Normalized Index of the NSWIR domain for SMC estimation from Linear correlation [9]) and NINSON (Normalized Index of NSWIR domain for SMC estimation from Non-Linear correlation [9]):

(6) WISOIL=R1450/R1300

(7) NSMI=(R1800−R2120)/(R1800+R2120)

(8) NINSOL=(R2080−R2230)/(R2080+R2230)

(9) NINSON=(R2120−R2230)/(R2120+R2230)

We chose to take the opposite of NINSOL and NINSON and termed them RNINSOL and RNINSON (R stands for “revised”) to create indexes that increase with moisture content. CAI (Cellulose Absorption Index) was proposed to discriminate senescent vegetation (e.g., crop residues) from soil [50,51]. It leverages the cellulose–lignin broad absorption feature at 2.1 µm:

(10) CAI=0.5(R2000+R2200−2R2100)

Interestingly, this feature progressively disappears when the water content in the senescent vegetation increases; it is absent in fresh vegetation [51,52]. On the other hand, CAI is negative in bare soils and vanishes when moisture content increases. In summary, a CAI close to 0 suggests the presence of water, in soil or vegetation, whereas high absolute values indicate the presence of either dry vegetation (CAI > 0) or dry bare soil (CAI < 0). This observation suggests that the absolute value of CAI, maybe normalized (i.e., n|CAI|), could be used as a water leak indicator.

The plant senescence reflectance index (PSRI) involves only spectral bands in the visible spectrum; it was proposed to determine the stage of leaf senescence through the increase in the ratio of carotenoids to chlorophylls [53,54]:

(11) PSRI=(R678−R500)/R750

Similar to CAI or n|CAI|, local variations of PSRI could be an indication of leaks.

**Figure 2:**
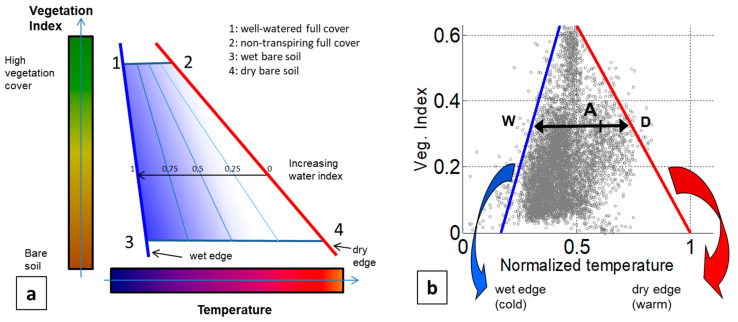
(**a**) Principle of the Triangle/Trapezoid Method with an interpretation of the four vertexes of the T-VI scatterplot (adapted with permission from [24,26]. Copyright 2022 Elsevier). The “dry” and “wet” edges are the “hot” and “cold” (here linear) boundaries of the scatterplot, respectively; they define the 0 and 1 isopleths of the water index. Three other isopleths were added, which correspond to 0.25, 0.5 and 0.75 water index levels. (**b**) Typical 2D scatterplot of (normalized) temperature and vegetation indices obtained during the first campaign.

In the Triangle/Trapezoid Method, the temperature signal is associated with a vegetation index (VI), which is used as a proxy for the vegetation cover-fraction. The relative position inside the 2D T-VI scatterplot of the representative point of a given pixel provides information on the water availability or evaporation rate at this pixel (Figure 2, [24,25,26,27,28,29,30,31,32,33,34,35,36]). Due to its ease of implementation, we adopted the empirical approach proposed by Sandholt [26] generalized to the case of a non-isothermal “cold” (i.e., “wet”) edge. In this method, two edges are drawn, respectively on the “cold” side (i.e., the “wet” edge) and the “warm” side (i.e., the “dry” edge) of the experimental T-VI scatterplot. Then, for each VI-level class, a linear water-index (WI) scale is defined which spans between the points of the “wet” and “dry” edges at the considered VI value. Hence, the water index at a pixel where the temperature is measured to be $T_{A}$ is obtained from (see Figure 2b):

(12) $$\mathrm{WI}=\left(T_{dry}-T_{A}\right)/\left(T_{dry}-T_{wet}\right)$$

where $T_{dry}$ and $T_{wet}$ are the temperatures read on the respective “dry” and “wet“ edges at the same VI-level as the considered pixel. Thus, the water index corresponds to the relative distance from point A to the “dry” edge, at iso- level of vegetation-index.

How to set the so-called “dry” edge and “wet” edge on both sides of the 2D scatterplot (Figure 2b) can receive multiple answers (e.g., [31,33,34]). Most often, these two edges are drawn as straight lines that should be set as close as possible to the 2D scatterplot, from respectively the high-temperature side, and the low-temperature side. A few outliers may be present at the boundary of the scatterplot which correspond, for example, to buildings, roads, and water surfaces, or originate from instrumental noise. These outliers should be rejected, which explains that a compromise has to be found between the proximity of the edges with the scatterplot and the number of points rejected outside the area between the two edges. The linear edges can be obtained by performing a linear regression of minima and maxima percentiles for each VI-class [33,34]. The percentiles cannot be set *a priori*; the percentiles that best fit the in-situ data must be determined each time [35]. The method we applied to define the linear edges is based on an optimization problem. It consists in finding for each of them the slope and intercept that minimizes the compound distance

(13) $$d=\sum_{i\in A}\left|T_{i}-T_{l_{i}}\right|+k\sum_{i\in R}\left|T_{i}-T_{l_{i}}\right|$$

where A and R are the sets of accepted and rejected points, respectively, $T_{i}$ is the temperature corresponding to point i, $T_{l}{}_{i}$ is the temperature read on the edge at the same VI as point i, and k is a penalization factor amplifying the distance of rejected pixels. Satisfying results were obtained by setting the penalization factor k in the range of 50. In some cases, this automatic adjustment of the edges led to visually unacceptable results; the edges were then corrected manually.

Regarding the VI involved in the Triangle/Trapezoid Method, a classical choice is to use NDVI (Normalized Difference Vegetation Index) which corresponds to the normalized difference between the spectral reflectances in the red and the near-infrared (typically at about 0.65 μm and 0.8 μm, i.e., on each part of the red edge related to chlorophyll). This index is strongly correlated with the vegetation cover-fraction: it ranges from about 0.1 for bare soil to values close to 0.8 for dense and green vegetation, depending on the wavelength choice. However, NDVI is sensitive to the soil’s spectral characteristics. OSAVI (Optimized Soil Adjusted Vegetation Index) [55] is less dependent on soil background and therefore was also used here:

(14) OSAVI=(R860−R660)/(R860+R660+0.16)

More recently, a variant of the Triangle/Trapezoid Method, the Optical Trapezoid Method, was proposed for sensing soil moisture where the temperature is replaced by a transformed reflectance obtained from a single SWIR reflectance measurement, either at 1.6 µm or 2.2 µm [56].

**Figure 3:**
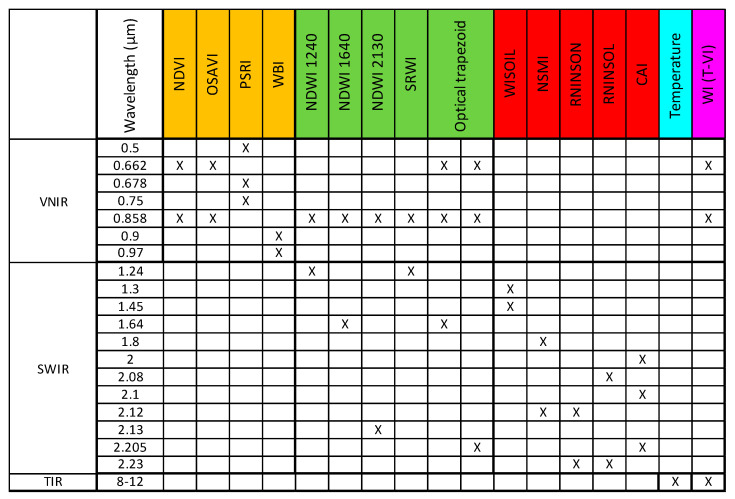
Considered spectral bands, spectral indexes and multiwavelength methods. The combinations involving wavelengths in the visible to NIR range are in orange, those with wavelengths from the visible to SWIR range are in green, those with wavelengths in the SWIR range only are in red, and the T-VI method is in purple. The use of the thermal infrared signal alone was considered as well (blue case).

The spectral indexes and constituent spectral bands that are considered herein are reported in Figure 3. The objective was to compare the performances of several multiwavelength methods in detecting areas of abnormal moisture which could be related to water leaks. The use of two hyperspectral cameras allowed us to explore different wavelength combinations from the visible to 2.5 µm, either on a standalone basis or possibly joint to the thermal infrared signal.

### 2.2. Results

The preliminary analysis according to the methodology described above was performed in February, April and July 2017 on several sites of the water network infrastructure belonging to SCP with variable weather conditions. In the first site, Esparron, which contains meadow, fallow and vineyards, a high moisture area was noticed near a pipe.

**Figure 4:**
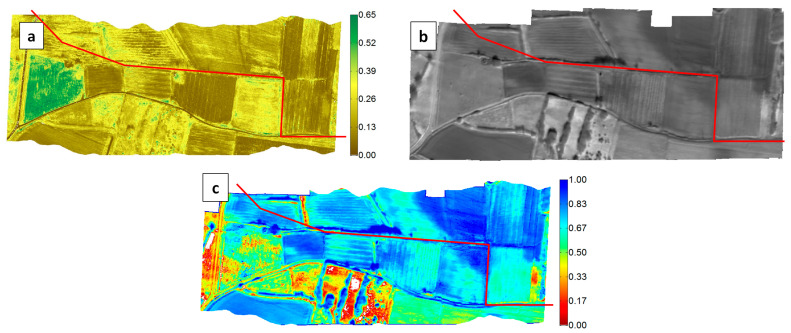
Results obtained over the Esparron test site on 16 February 2017. (**a**) Map of the Vegetation Index OSAVI. (**b**) Thermal Infrared mosaic (TIR) (dark/clear pixels correspond to low/high brightness temperatures). (**c**) Inferred map of the water index WI according to Equation (1) (the underlying 2D scatterplot of the normalized brightness temperature vs. Vegetation Index is in Figure 2b). The pipe position is shown in red in the three images.

The images involved in the T-VI Triangle/Trapezoid Method are shown in Figure 4. OSAVI was used for the vegetation index because of its higher robustness with respect to bare soil optical variability, but the results obtained with NDVI were very close. During the flight in February 2017, vegetation was either absent, senescent or still dormant. Thus, the OSAVI map presents quite low values, except over trees and one particular field on the left (Figure 4a). Interestingly, the thermal infrared image (Figure 4b) presents in the top-right part a colder area of triangular shape. It corresponds to a depression running towards the top of the image where stalled water had occasionally been detected over the past years.

**Figure 5:**
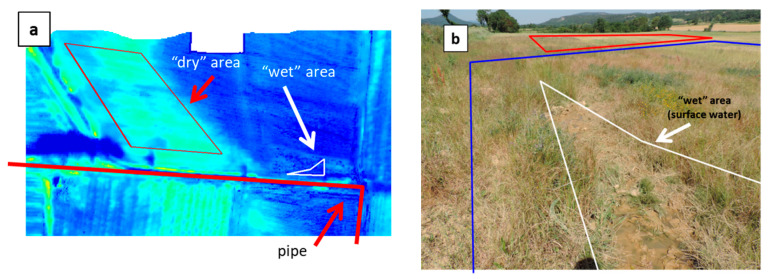
(**a**) Close-up of the top right part of the water index map in Figure 4c, a “dry” area chosen for reference and a “wet” area showing surface water. (**b**) Picture showing the “wet” area in the foreground and the “dry” area (red polygon) in the background (picture taken on 12 June 2017). The blue polygon in Figure 5b refers to a soil depression where it is believed that water spurting from the soil in the “wet” area may runoff, hence leading to high WI values.

The cold (blue) and warm (red) edges of the T-VI scatterplot have been positioned so that the trapezoid pattern contains essentially all the scatter, except for maybe a few outliers (Figure 2b). The inferred Water Index (WI) is in Figure 4c, where a high value of WI (in blue) means that the soil moisture is at the highest level for the considered VI level. The triangular area with a low temperature in Figure 4b is related to high WI values (WI > 0.84). A close-up is reported in Figure 5a where the closest water pipe location has been added. Two areas have also been drawn to help interpret the results. The so-called “wet” area, which is in a meadow field, corresponds to a very humid zone located just north of the pipe, not far from a 90° turn, with mud and even a puddle with spurting water from the soil (see the picture in Figure 5b). A “dry” area (red polygon) was selected for reference in a well-drained field just uphill to the west that was also covered with a meadow (Figure 5b).

**Figure 6:**
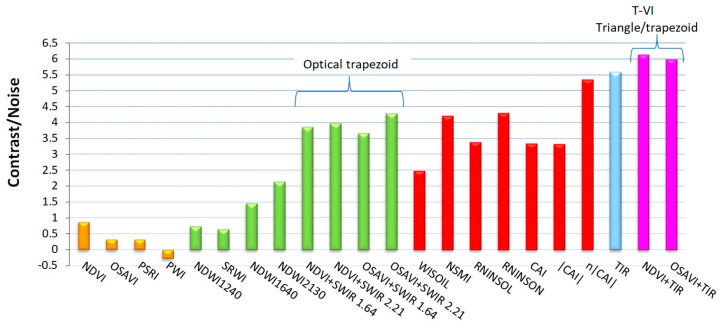
Contrast-to-noise (C/N) ratio when comparing the signal over the “wet” area and the reference “dry” area. The information is extracted from index maps based on optical signals (reflectance) in the VNIR spectrum (orange bars), in the VNIR + SWIR spectra (green bars), in the SWIR spectrum alone (red bars), or from the raw thermal infrared signal (TIR) radiance temperature (blue bar), or by combining VNIR + TIR spectra as in the T-VI Triangle/Trapezoid Method (purple bars). Same colors as in Figure 3.

The WI was analyzed in the “wet” and “dry” areas to produce average values and standard deviations. Therefrom, a contrast-to-noise ratio (C/N) was calculated (the contrast between “wet” and “dry” areas divided by the mean standard deviation). The same was performed with the other spectral indexes listed in Figure 3 and with the radiance temperature alone, i.e., the raw thermal infrared signal; the results are summarized in Figure 6.

Indexes based on VNIR signals alone led to very poor results (orange bars): the signal variability (standard deviation) in the “dry” and “wet” areas was actually higher than the mean difference (contrast), which gave a contrast-to-noise ratio lower than 1. The contrast seen in the PWI image was even negative, however, this has little significance since the PWI values on the “dry” and “wet” areas were close to one another, namely 1.09 and 1.07, but presented a correspondingly high standard deviation of resp. 0.06 and 0.08. Better results were obtained when combining VNIR with SWIR (green bars) or when using SWIR signals alone (red bars). For example, a C/N higher than 3 was obtained with the following indexes: NSMI, RNINSOL, RNINSON, CAI, or with the “Optical Trapezoid Method” described in [56]. However, the best results (i.e., C/N > 5.5) were obtained with the thermal infrared data, either by considering the raw radiance temperature image alone (C/N = 5.6) or, a little better, through the Triangle/Trapezoid Method by combining it with NDVI (C/N = 6.1) or OSAVI (C/N = 6). In summary, the strongest signature of soil moisture was in the TIR image. The Triangle Method still improved the results slightly, however, using OSAVI or NDVI for the T-VI scatterplot was quite equivalent.

The pipe in the Esparron area was finally checked for leaks in June with ultrasound sensors. The soil was dug in several places before and after the 90° turn in order to put the microphone directly on the pipe, but no leak was found. The conclusion is that the water seen in the “wet” area must have a natural origin (groundwater exfiltration). Nevertheless, the consequence on the soil is of the same nature (i.e., moisture increase and runoff) as with a pipe leak; the quantitative results obtained by processing the hyperspectral and infrared thermal data (Figure 6) are thus thought to be representative of the performance expected for leak detection.

**Figure 7:**
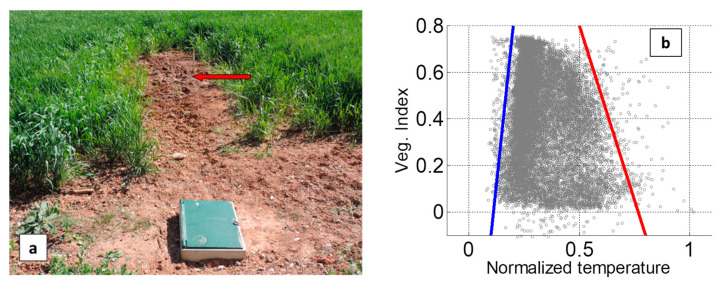
(**a**) Picture (directed southward) of the Le Tholonet test site (spring-wheat field) where an artificial leak was introduced. To the main pipe running east–west, an additional pipe was connected (see the connection box) in the south direction. The added pipe had a 10 mm hole, 5 m south of the connection (as indicated by the stick and the arrow in the gap downhill). (**b**) 2D scatterplot of temperature and vegetation index as obtained during a flight on 21 April 2017.

In the second test site, Le Tholonet, an artificial leak was introduced by connecting to the existing pipe (grey cast iron DN 80), at a depth of about one meter, a pipe of high-density polyethylene DN 50. This new pipe apparatus included a valve, a flow meter, a recorder and a pressure regulator. A hole 10 mm in diameter was drilled on the side of the additional pipe, 5 m from the connection, yielding a 180 L/h water flow (Figure 7a). A reference zone with a similar dig-work but without any buried pipe was added about 15 m away (in the eastern direction) to discriminate between dig-work effects and leak effects.

**Figure 8:**
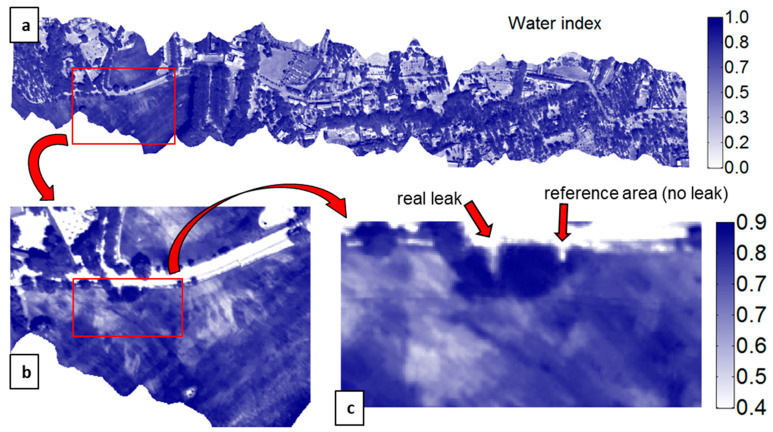
Water Index over the Le Tholonet area obtained on 21 April 2017. (**a**) General view corresponding to a single flight line. (**b**) Close-up of the WI map showing the artificial leak in a wheat field, south of the road in white. (**c**) Still closer view of the WI map showing two strips of bare soil, one over the leaking pipe (on the left) and the other one acting as reference (bare soil in the wheat field, without leak). The Water Index reaches highest values in an area surrounding the leak and diffuses east to the reference area (dark-blue half-disk).

The 2D scatterplot of normalized radiance temperature vs. vegetation index (OSAVI) based on a flight performed on 21 April 2017 is reported in Figure 7b and the inferred WI map is in Figure 8a. The test site is located in a peri-urban area with scattered tall vegetation (trees) responsible for the high heterogeneity of the WI image. A very small number of outliers are observed with negative values of OSAVI. This can be explained by the combined effect of high heterogeneity in surface type (thus in terms of visible and near-infrared reflectances), and slight errors (i.e., pixel-size) in image registration, which makes the OSAVI calculation able to involve the visible reflectance of one spot and the near-infrared reflectance a neighbor spot instead of, theoretically, the reflectances of a single spot. Care has to be taken not to mistake trees and their shadows for leak signatures. Nevertheless, the artificial leak introduced in the north edge of a spring-wheat field (in the middle of the red rectangle in Figure 8a) generates a significant contrast as highlighted in the close-up, as shown in Figure 8b,c. The FDR probe indicated a soil moisture of 30%, 48%, 45% and 34% at, respectively, +3, +1, −1 and −3 m in the northward direction from the leak (the connection to the main pipe is at +5 m); while the moisture is only 6% at 1 m, west of the connection. Although the WI scale is only loosely related to the real moisture content, these results are coherent with the distribution of the WI contrast in the leak area.

Overall, the methodology was proven to be useful for leak detection and the wavelengths were determined for the selection of the multispectral cameras to be used in operational leak detection.

## 3. Development of an Operational Service Based on MAV and UAV

### 3.1. Materials and Methods

After the preliminary assessment analysis, the objective was then to validate the T-VI (Temperature–Vegetation Index) method at different scales by using two platforms: a manned air vehicle (MAV) and an unmanned air vehicle (UAV).

**Figure 9:**
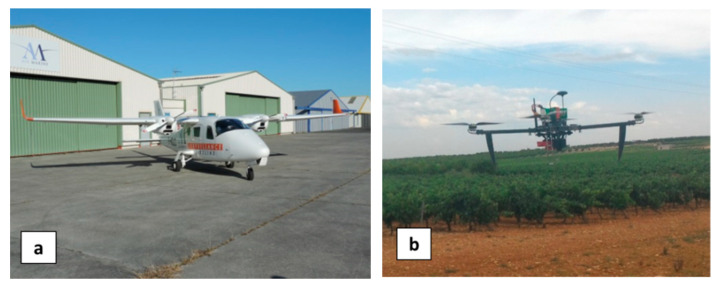
(**a**) Airplane platform (MAV) and (**b**) UAV platform equipped with two specific multispectral sensor systems.

The aircraft (MAV) was a Tecnam P2006T (Figure 9a) instrumented with a Spectrocam (Pixelteq, Largo, FL, USA) VNIR multispectral camera with a filter-wheel (filters at 660 nm and 832.5 nm) and a Noxcam 640L (Noxant, Palaiseau, France) thermal infrared (TIR) cooled camera (7.7–9.3 µm), all on a rigid camera platform. The flights with the manned platform were performed at a typical altitude of 800 m which led to a spatial resolution of 0.30 m for the VNIR camera and 0.48 m for the TIR camera.

The UAV was a custom-designed multicopter (Figure 9b) instrumented with a RedEdge 3 (Micasense, Seattle, WA, USA) VNIR multispectral camera (with five bands: 475; 560; 668; 717; and 840 nm) and a microbolometer uncooled Vue Pro R (FLIR, Portland, OR, USA) TIR camera (7.5–13.5 µm). The flights with the unmanned platform were performed at a typical altitude of 50 m which led to a spatial resolution of 3.4 cm for the Micasense camera and 6.5 cm for the FLIR camera.

Three bands were used in both platforms: 660 nm (red), 832.5 nm (NIR) and TIR for manned aircraft, and 668 nm (red), 840 nm (NIR) and TIR for UAV. A radiometric calibration based on a Spectralon target was applied as part of the radiometric calibration carried out by Pix4D in the case of the red and NIR bands of the UAV acquired data to get spectral reflectances. On the other hand, the multispectral aircraft data were uncalibrated raw data. In addition, although the aircraft and the UAV TIR cameras were both radiometrically calibrated, no correction was applied to extract the true temperature from the brightness temperature (as obtained by setting the emissivity parameter to 1 and applying no atmospheric compensation). Actually, since the Triangle/Trapezoid Method is founded on a normalized temperature (namely a ratio of temperature differences) and the main role of the vegetation index is to be a discriminating factor accommodating a linear bias, negligible differences were observed in the resulting wetness index whether using calibrated or uncalibrated remote sensing data (nonetheless, the use of OSAVI requires access to reflectance values as opposed to NDVI which, to first approximation, accommodates uncalibrated spectral data).

The images from both platforms were pre-processed with the photogrammetric software Pix4D to obtain spectral orthomosaics, which were then co-registered by Wadileaks, the custom processing software developed in the frame of the WADI project [57]. The selected photogrammetric software Pix4D makes the co-registration of the two bands from the multispectral camera on the UAV flawless. However, when applied on orthomosaics obtained with the cameras used on the MAV, the co-registration did not achieve the same success. Gefolki software [41] had to be implemented to solve the co-registration errors. In both cases, the co-registration was done by taking as master the TIR image, which is the image with lowest resolution; for this purpose, the VNIR images were resampled with a bi-cubic spline interpolation algorithm.

### 3.2. Results of a First Leakage-Detection Campaign with MAV and UAV (France)

Data were first acquired in October 2018 over several water network districts operated by SCP in France. The scheduled flights took place four days after a rainy period. In addition, the sky was often cloudy during the intermediate days, reducing the evaporation and transpiration of the accumulated water. Most likely the soil still contained quite a lot of water on the day of the flight, reducing the difference between soil moisture induced by leaks (natural or artificial) and soil moisture in the surroundings. Indeed, in some test fields, water puddles were spotted as remnants of the recent rain.

**Figure 10:**
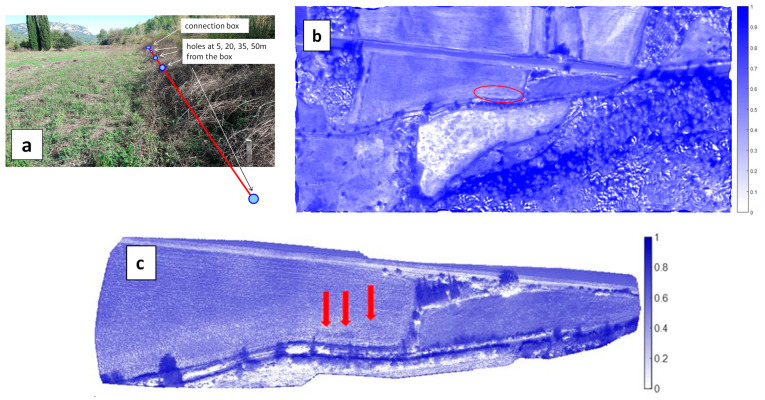
(**a**) Picture of the Vauvenargues test area with artificial leaks. Picture taken towards the east from leak n°4 (stick in the bottom right of the image) with the connection box in the background, in front of the trees. The leaking pipe (red line) is running from background to foreground at the limit between the high-grass area in the south and the low-grass area in the north. (**b**) WI map in the Vauvenargues test area obtained on 22 October 2018 with the MAV. The leaking pipe is along the long axis of the red ellipse; (**c**) same with the UAV. Spots with faint contrast close to the leaking pipe are shown with red arrows.

In the Vauvenargues test area, a new pipe of high-density polyethylene was connected to the existing (steel) pipe and buried at a depth of about 1 m, more than one year before the flights. The connection with the existing pipe was equipped with a valve, a flow meter, a recorder and a pressure regulator. Four calibrated holes were drilled in this new pipe to generate leakages. They were at 5 m (10 mm diam.), 20 m (10 mm diam.), 35 m (5 mm diam.) and 50 m (5 mm diam.) from the connection box (Figure 10a).

On the day of the flight (22 October 2018), the soil moisture content was measured with a portable IMKO TDR probe with 16 cm rods. Moisture content in the field was in the range of 30–38% which is quite high. It increased significantly in the close vicinity of leaks n°1 and n°2 to reach 60% and more. Two small puddles (about 50 cm wide) could even be seen under the high grass. No moisture increase was however observed above the simulated leaks n°3 and n°4 which is partly due to a reduction of the hole diameter. In conclusion, the areas presenting increased moisture have a quite limited extent and were restricted around leaks n°1 and n°2. In addition, since the surrounding soil had a quite high moisture (between 30 and 40%), it was expected that the thermal contrast between the leak areas and the non-leak areas would be much lower than if the nominal soil was drier, as in summer or at least long after the last rain event.

The WI maps obtained with the MAV and UAV do not reveal a significant contrast along the leaking pipe, except in some faint spots, not exactly corresponding with the actual leaks n°1 and n°2 (Figure 10b,c). These results suggest that for successful leak detection it is necessary to select a time of year when the difference in evaporation flux between a leak area and its surroundings is maximized.

A rigid camera platform was used onboard the MAV for the airborne campaigns in France; later, for the campaigns performed in Portugal, it was replaced by a 2-axis gyrostabilized platform which contributed to obtaining images of better quality.

### 3.3. Results of a Second Series of Leakage-Detection Campaigns with the MAV and UAV (Portugal)

Two airborne campaigns were performed in May and September 2019 over a series of water network districts operated by EDIA in Portugal. The objective was to validate the findings obtained during the campaigns in France, to assess the qualitative gain of the images provided by the gyrostabilized platform, and to improve the leak-detection results by selecting more appropriate meteorological conditions. The first campaign was over the Monte Novo block, near Sao Manços, while both the Vale de Gaio and Ferreira do Alentejo areas were added into the second campaign. Artificial leaks were built with different scenarios: surface or underground leaks, single or repeated events, surfaces with bare soil, and low- or high-grass areas. Furthermore, areas with known water-flow events were included in the MAV/UAV flight zones of the two campaigns to help interpret the origin of these flows. Additionally, an acoustic campaign was performed prior to the flights in Monte Novo to detect and locate any current leaks. A large amount (93%) of the 130 km Monte Novo pipe network is made of concrete or high-density polyethylene (PEAD) whereas about 6% is ductile iron and less than 0.5% is steel (classic sound-based methods are less efficient on concrete and plastic pipes than on metallic pipes).

**Figure 11:**
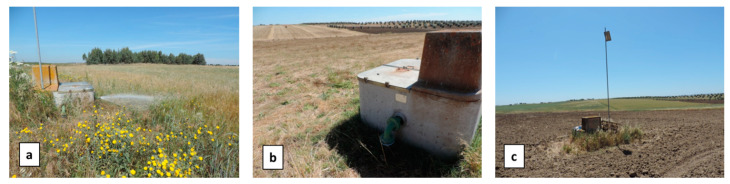
Pictures in May 2019 of (**a**) hydrant H5.6, (**b**) hydrant H10, (**c**) hydrant H11 and the corresponding environment, respectively, a meadow with high grass, a meadow with short dry grass, and bare soil. The picture in (**a**) was taken while the valve was open to simulate a leak.

Three artificial leaks were produced at hydrants H5.6, H10 and H11 (Figure 11). Virtual leaks were essentially produced by simply opening the vane of the hydrant for several periods ranging from 10 min to 1 h during the three days of the flight campaign.

**Figure 12:**
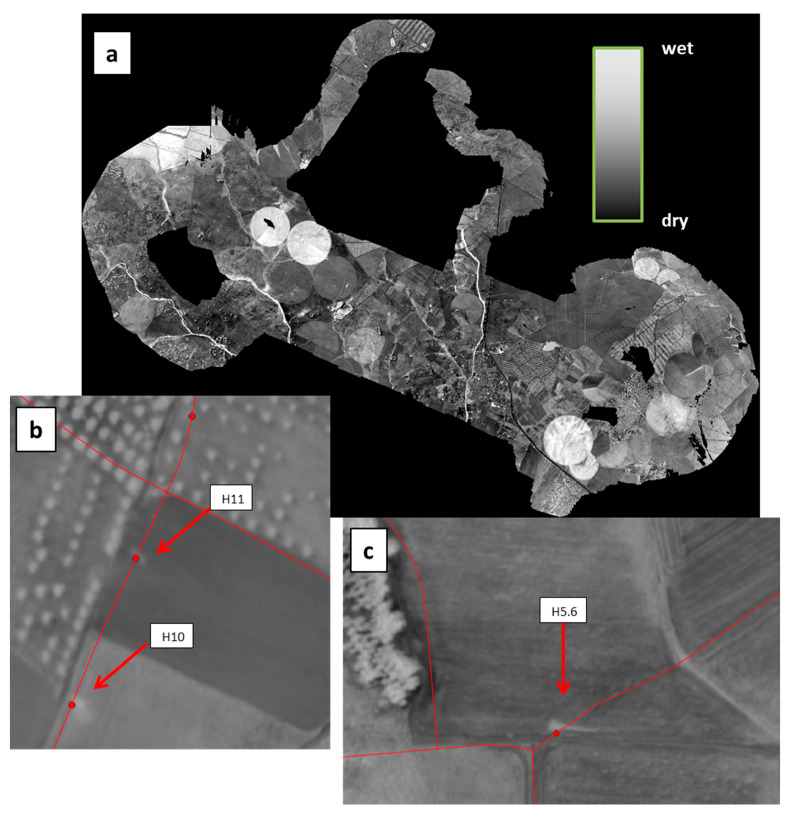
(**a**) WI map obtained on 16 May 2019 with the MAV over the Monte Novo district around São Manços, Portugal. The covered area is about 2 km × 10 km. (**b**) Detail of the WI image overlaid with the water network (in red) showing hydrants H10 and H11. (**c**) Same with hydrant H5.6. The three vanes were opened for periods from 10 to 60 min during the previous three days to simulate leaks.

The MAV flights in May and September were performed at 1200 m altitude, leading to a Ground Sample Distance (GSD) of 0.42 m and 0.73 m for the multispectral and thermal infrared cameras, respectively. The flight lines during the 16 May operation and the resulting Water Index map can be seen in Figure 12a. The village of São Manços can be seen in the central part of the image; the remaining surface is essentially rural with several fields having central pivot irrigation. A local increase of the water index can be seen in the region of the artificial leaks H5.6, H10 and H11, precisely in the direction of each water jet (Figure 12a,b).

**Figure 13:**
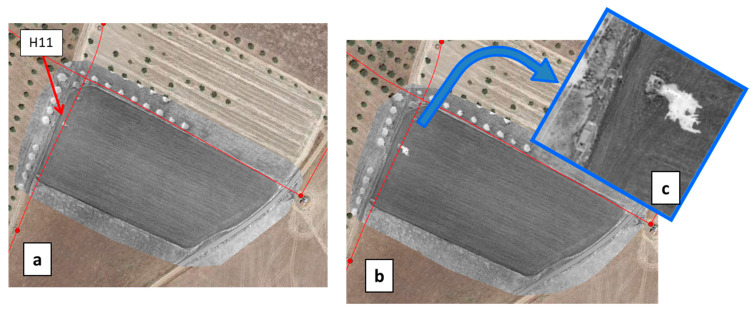
WI images obtained with the UAV on 14 May 2019 over the test field containing hydrant H11, (**a**) before and (**b**) after the vane was opened to simulate a leak; (**c**) is a close-up over the hydrant area. The WI images are in greyscale (white for wet and black for dry), the water network is overlaid in red and Goggle Earth data (in color) fill the surrounding area. Covered area is about 130 m × 240 m. The contrast between wet and dry soil is very high.

**Figure 14:**
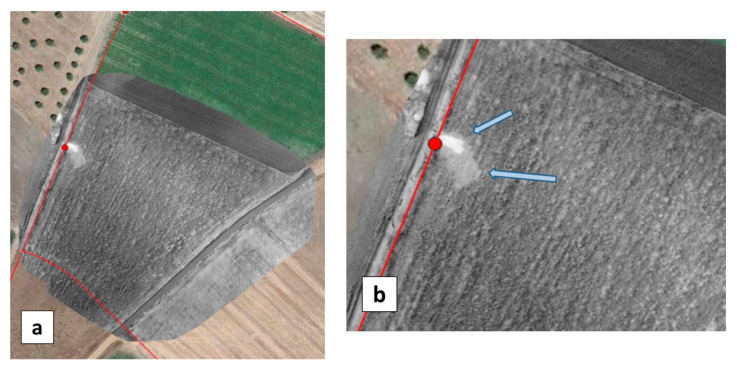
(**a**) WI obtained with the UAV on 16 May 2019 in the test field containing hydrant H10. The WI image is in greyscale (white for wet and black for dry), the water network is overlaid in red and Google Earth data (in color) fill the surrounding area. (**b**) Close-up of the WI image with arrows indicating two contrasted areas (see text).

**Figure 15:**
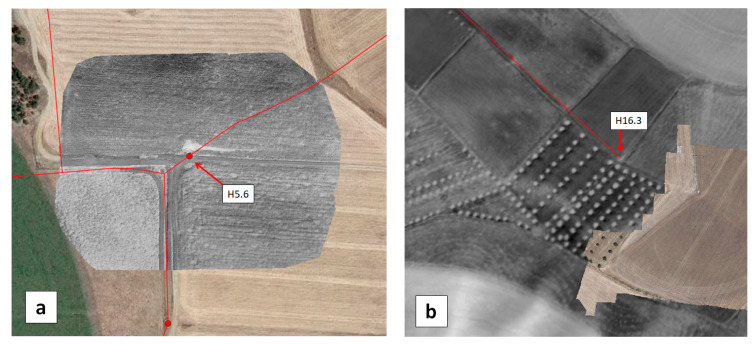
(**a**) WI images obtained with the UAV on 16 May 2019, over the test field containing hydrant H5.6. The WI images are with greyscale (white for wet and black for dry), the water network is overlaid in red and Goggle Earth data (in color) fill the surrounding area. (**b**) Excerpt of the WI map obtained on 16 May 2019 with the MAV in the vicinity of a small leak at hydrant H16.3 that was previously detected by the acoustic method.

Significant contrasts were also obtained with the UAV (Figure 13a, Figure 14a and Figure 15a). Thanks to a higher spatial resolution due to the lower flight height (resolution was 7 to 9 times better than with the MAV), the UAV cameras could even make the distinction between a recent leak event and former leak events that occurred in the three previous days (Figure 14). The upper arrow in Figure 14b indicates a small area of high moisture resulting from the vane opening on the same day. The lower arrow indicates an area of larger extent, turning south and encompassing the former area, and showing lower moisture values (but still higher than the rest of the field); this is the result of former vane openings performed during the previous three days. With regards to the question of whether the WI contrast resulted from a temperature contrast or a VI contrast, we can state that in the case of bare soil conditions (H11), a contrast related to water presence was revealed in both OSAVI and TIR images (although with higher strength in the TIR image), whereas, in the presence of vegetation (H5.6, H10), a contrast was observed essentially in the TIR image alone (not shown here).

The optical data complied with moisture measurements. At the time of the May 2019 flights, soil moisture content near hydrant H10 showed a sharp decrease from 70% at 1 m distance to 41% at 6 m distance and then 34% at 11 m distance. Similarly, soil moisture was 38%, 35% and 31% at distances of, respectively, 1 m, 6 m, and 30 m from hydrant H5.6 in a narrow strip in the direction of the water jet (elsewhere the soil was too dry to use the TDR moisture probe). Around hydrant H11, before opening the vane, the soil moisture was in the range of 5–8%. At the time of flight, i.e., 2.5 h after the leak event (vane opened for 30 min) moisture was in the range 34–36% up to 8 m distance from the hydrant and then dropped to 10% at 13 m and 7% at 18 m.

In the immediate vicinity of H16.3 (where the acoustic campaign detected a small superficial leak in a tube on the hydrant), the WI image obtained with the MAV reveals a slight moisture contrast (Figure 15b). However, this signal is quite faint and could be confused with the signal originating from the changes in vegetation type and height commonly observed close to the hydrants.

**Figure 16:**
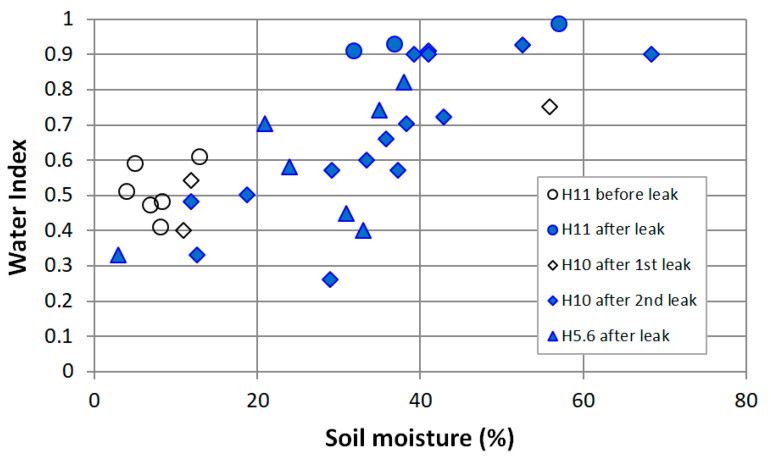
Correlation between the local Water Index inferred from the multispectral images obtained with the UAV and the soil moisture measured with a TDR probe at distances ranging from 0 to 30 m from the artificial leaks at hydrants H10, H11 and H5.6 (Figure 11, Figure 12, Figure 13, Figure 14 and Figure 15a).

Over 160 soil moisture measurements were performed with hand-held probes in the test fields discussed in this paper. The purpose was essentially to help monitor the artificial leak flow and interpret the UAV and MAV results. The leaks had a quite small spatial extent, nevertheless, thanks to the high spatial resolution of UAV data, a quantitative analysis of the interplay between soil moisture and Water Index could be performed, based on the remote sensing results obtained around the leaks introduced at hydrants H10, H11 and H5.6 (Figure 16). Although the data in Figure 16 present an important scatter, which is partly explained by the measurement error of the TDR probe and by the soil moisture variability at the decimeter scale, the positive correlation between soil moisture and WI is confirmed. The coefficient of correlation (r^2^) reaches 0.56.

**Figure 17:**
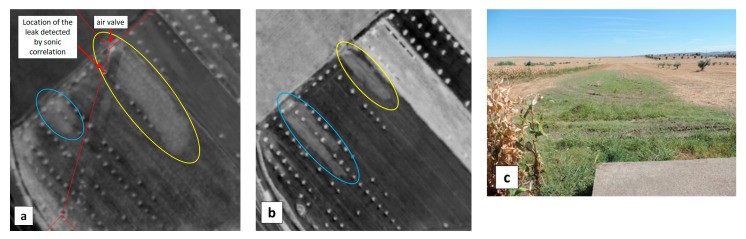
Excerpts of the WI maps obtained with the MAV (**a**) on 16 May 2019, (**b**) on 11 September 2019 in the vicinity of a real leak previously detected by sonic correlation, 26 m south of air valve VS13.1VS9. (**c**) Picture taken on 10 September 2019 from the air valve, in the southeast direction; the WI anomaly in the yellow ellipse in (**b**) corresponds to the green grass area extending downhill from the air valve (located in the concrete block in the foreground).

The acoustic campaign in Monte Novo located another leak by sonic correlation at 26 m from an air valve. Close to it, the WI image showed a significant contrast over an elongated area starting from the air valve and extending downhill in the southeast direction (yellow ellipse in Figure 17a). A similar pattern, yet shorter, was observed in the same field (blue ellipse in Figure 17a). During the next flight on 11 September 2019, the first pattern shrunk whereas the second one appeared more elongated (Figure 17b). The second anomaly was ruled out as a leak because its location was mainly north, i.e., uphill, of the pipeline (Figure 17a). A ground-truth measurement on 10 September 2019, showed that the mean moisture level was about 13% along the first WI-anomaly while reaching up to 23% locally (yellow ellipse in Figure 17b), whereas it was only about 7% a few meters away. Sparse green vegetation (short grass) could be seen along the WI anomaly, as opposed to dry vegetation away from it (Figure 17c).

**Figure 18:**
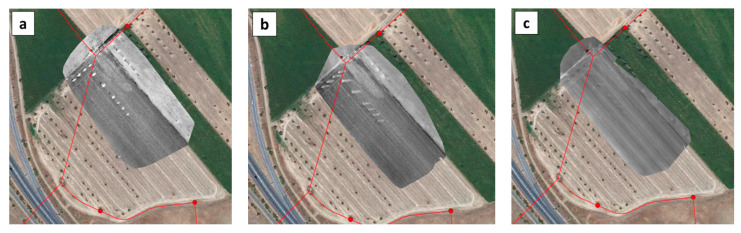
WI images obtained on 10 September 2019 with the UAV, of a field in the Monte Novo area having shown a “water anomaly” in the MAV images recorded during the April and September 2019 campaigns (Figure 17). The WI anomaly is delineated with a yellow ellipse in Figure 17b. WI images were obtained at (**a**) 16:01 h, (**b**) 18:11 h, and (**c**) 20:00 h, just after sunset.

The UAV was operated on the same site on 10 September 2019 for a multitemporal analysis. WI images were obtained at 16:01 h, 18:11 h, and at 20:00 h, just after sunset (Figure 18).

During the late afternoon, the temperature of moist and dry areas decreases differently, which makes the contrast over the WI anomaly appear at a maximum in the 18:11 h image (Figure 18b). Nevertheless, at such a late time in the day, one faces adverse effects like tree shadows (notice the left-to-right, i.e., west-to-east, bright strips emanating from each olive tree in Figure 18b). The present observation regarding the time evolution of the WI cannot be generalized since the area considered by the UAV was too small for building a well-populated scatterplot as required in the Triangle/Trapezoid Method.

Soil was later dug out to find out if the apparent increase of soil moisture could be related to a water leak stemming from the nearby pipe or air valve. Deep soil was however found to be dry. The WI anomalies in Figure 17 and Figure 18 were finally attributed to an excess of irrigation in the uphill field thus leading to recurring runoff into the downhill field.

**Figure 19:**
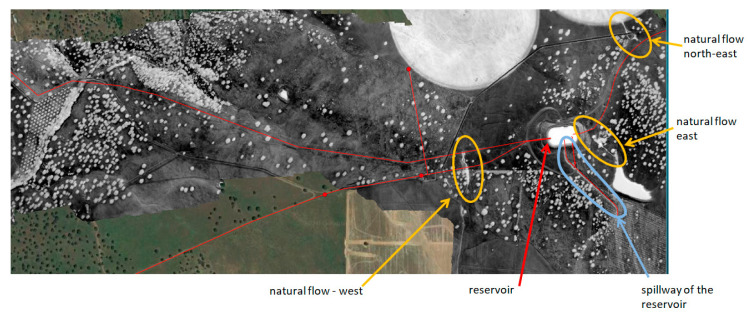
WI image obtained with the MAV on 11 September 2019, in the Vale do Gaio district, Portugal, with the Barras water reservoir. The spillway of the reservoir can be seen in the southeast, together with three “water anomalies” which, after ground inspection, were actually explained as natural water flows. Other WI positive signatures originate from trees (scattered small circles) and two irrigated fields (large white disks in the northeast of the image).

The Barras water reservoir is located in the district of Vale do Gaio and three water-flow anomalies west, east and northeast of the reservoir had been suspected to be leaks. These anomalies have modified the vegetation cover and hence the surface optical properties (short green grass and small puddles as compared to dry grass or dry bare soil elsewhere). The MAV and UAV flights were performed over the reservoir zone to assess the detection performances. Indeed the MAV optical system detected the three water-flows west, east, and northeast of the reservoir, as well as the spillway (Figure 19).

**Figure 20:**
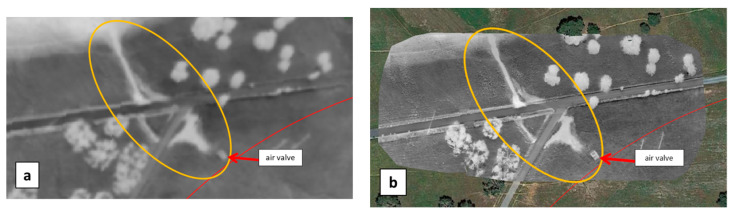
(**a**) Close-up of the WI image in Figure 19 over the northeast “water anomaly”. (**b**) WI image obtained in the same area as the UAV on 11 September. (FOV: 83 m × 67 m). Water is flowing in northwest direction from the concrete block containing an air valve and splits in two arms (light grey marks in the yellow ellipse). The two arms run under the road and join downstream before reaching a field with central pivot irrigation in the north.

The UAV optical system provided WI images with an improved spatial resolution in smaller sensed areas (compare Figure 20a,b).

After the airborne campaign, EDIA dug the soil close to the air valve northeast of the reservoir, at the origin of the surface water flow shown in Figure 20. The water flow was not attributed to a leak of the pipe running nearby. Water was found in the hole that EDIA dug. Then, for two weeks EDIA emptied the adductor and the reservoir but the water did not disappear in the hole, it kept the same level. A few days later, it rained and the level increased in the hole. The final interpretation is that the observed water is originating from the water table downhill of the reservoir. The same interpretation was given for the western and eastern water-flow anomalies. The eastern water-flow anomaly is a natural stream ending in a pond (see Figure 19).

**Figure 21:**
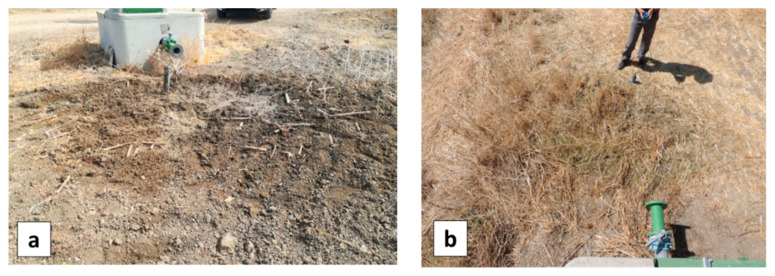
Artificial leaks in Ferreira district at (**a**) hydrant H9.7 (essentially bare soil) and (**b**) hydrant H16 (dry grass).

**Figure 22:**
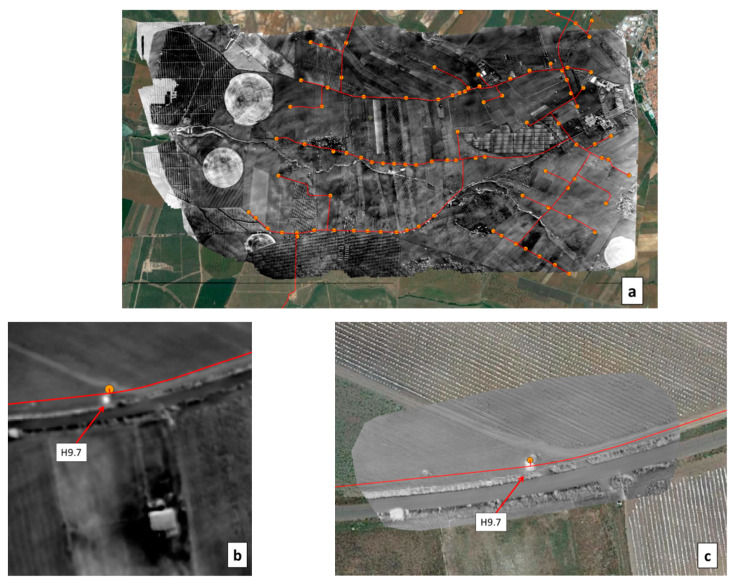
(**a**) WI image obtained with the MAV on 12 September 2019, over Ferreira district, Portugal, overlaid with the water distribution network (in red). (**b**) Detail of the WI image showing hydrant H9.7 and its artificial leak as obtained with the MAV. (**c**) WI image obtained with the UAV overlaid on a Google Earth image (in color).

**Figure 23:**
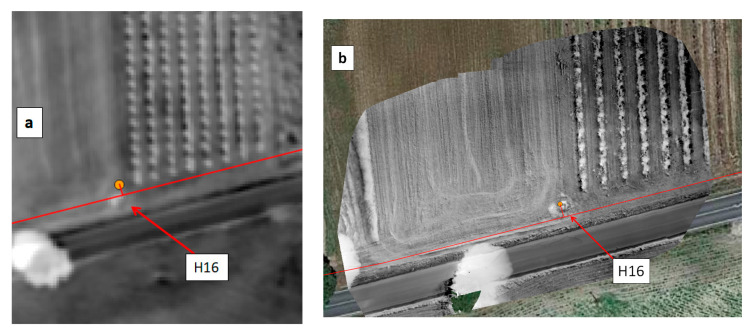
WI images (in greyscale) showing the artificial leak at hydrant H16 as obtained on 12 September 2019 with (**a**) the MAV (excerpt from Figure 22a) and (**b**) the UAV. The water distribution network is overlaid in red.

In the EDIA network infrastructure near Ferreira do Alentejo, underground artificial leaks were created at hydrants H9.7 and H16 by connecting to the existing pipe a plastic pipe with a hole and burying it (Figure 21). The MAV and UAV were flown on 12 September 2019 over this area. Figure 22a provides a WI map of the area that was covered by the MAV flight lines. After zooming in on hydrant H9.7 we get the representation in Figure 22b, which can be compared with the WI image obtained with the UAV system in Figure 22c. The same for the artificial leak at hydrant H16 is provided in Figure 23.

In all cases, the increased soil moisture induced by the artificial leak was clearly revealed in the WI image, although with higher contrast at H9.7 than at H16. This can be explained by the fact that the leak at H9.7 was under bare soil whereas at H16 the soil was covered with senescent grass. Moisture was in the range of 37% to 52% in the leak areas; it led to a temperature decrease as confirmed in-situ with an IR temperature sensor. At hydrant H9.7 the radiance temperature was 24 °C in the center of the moist area whereas it was in the range 40–44 °C far from the leak. At hydrant H16, the radiance temperature was up to 48 °C on bare soil, 44 °C on dry grass, 33 °C on green vegetation, and down to 30 °C on the moist senescent grass covering the leak area. In addition to the thermal effect, the leaks also had a consequence on the optical reflectance as could even be seen by the naked eye (see in Figure 21a,b).

A series of 16 anomalies (in addition to the two artificial leaks) can be identified in the WI image in Figure 22a by looking for local variations along every pipe and at each distribution node of the water network. They show a water index level substantially higher than the surroundings, which could be related to real leaks in the network. Among them, thirteen were observed on network parts accessible for in-situ evaluation. A first visit was made on 16 April 2020. The weather conditions were good, however, it rained around 30 mm during the previous 24 h. This rain event together with the long delay between the flights in September 2019 and the in-situ observation made it so that the actual soil occupation and soil moisture were totally different. In addition, due to the recent rainfall, water accumulation was found close to a number of hydrants because of their low position, hence precluding any unambiguous assessment. Another field visit was made on 25 June 2020 during a period of warm and dry weather.

Nine suspicious areas were discarded because no water accumulation was found around the considered hydrant or network structure. The explanation for the WI contrast nonetheless observed in September 2019 is that, due to previous rains and irrigation sequences, water was available for weeds, especially if the hydrant is at a lower point, and, due to the absence of herbicides or harrowing of the soil, vegetation inevitably took advantage of the situation, giving rise to a spot with reflectance variations and possibly a local increase in evaporation, which ultimately induced a WI increase. In the future, to avoid this type of false alarm, the WI threshold used for warning should be increased correspondingly.

**Figure 24:**
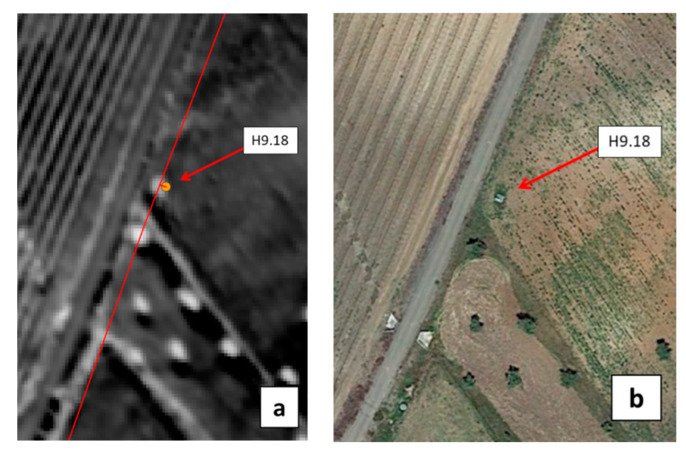
(**a**) WI image showing hydrant H9.18 as obtained with the MAV (excerpt from Figure 22a). (**b**) Google Earth image of the same area.

**Figure 25:**
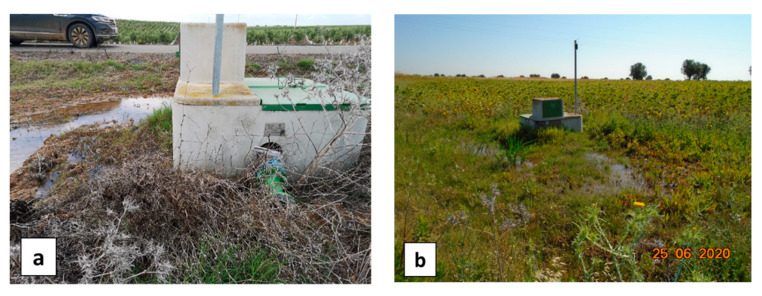
Pictures of hydrant H9.18 and surrounding vegetated ground taken on (**a**) 16 April 2020 and (**b**) 25 June 2020.

One detected spot actually corresponded to a soil depression close to a hydrant that was recurrently filled with water to water the cattle. Two other WI anomalies were attributed to a leak from the connection between the hydrant and the farmer’s infrastructure. Finally, a clear WI anomaly was observed at hydrant H9.18 (Figure 24a), where the first inspection on 16 April 2020 revealed a large puddle (Figure 25a). As the hydrant was used during the summer irrigation campaign, the detected WI anomaly might be explained by a leak from the connections between the hydrant and the farmer’s infrastructure. In addition, the hydrant is located at a low topography point which could have led to water accumulation at this spot, either from irrigation water before the remote sensing campaign in September 2019, or from rain, before the visit in April 2020. The next visit, in June 2020, showed again a large puddle around the hydrant (Figure 25b), yet the weather had been dry for a long time and the hydrant was not used for irrigation at this time. In addition, water leaking inside the hydrant’s concrete structure was clearly heard, definitely confirming that the WI anomaly revealed a leak.

Figure 24 also shows that just southwest of hydrant H9.18 is an olive tree with a WI even higher than that at the leaking hydrant. Since this tree is very close to the pipeline, the risk is high to mistake it with a leak in the pipe. Increasing the WI warning threshold above the contrast level at the tree has the deleterious consequence of missing the real leak at hydrant H9.18. This shows that not only the WI-distribution analysis should focus on the pipelines of the networks and the associated distribution structures (e.g., hydrants) but an in-situ inspection is mandatory to eliminate the unavoidable false alarms that appear if the WI warning threshold is maintained at a reasonable level chosen to not miss the most important leaks.

## 4. Performance Analysis and Discussion

A formal evaluation of the WADI service application is necessary to identify the conditions in which the proposed T-VI multispectral system will potentially have a greater chance of success in detecting water leaks. The performance of the T-VI multispectral system was thus analyzed based on 60 events from the flight campaigns performed in France and Portugal. The analysis was conducted using the processed images from UAV and MAV flights on both sites that were subject to ground-leak detection campaigns to identify potential water leak events. The analyzed leak events were: real leaks (23%), artificial leaks (30%), natural water flows (42%), or no leaks nor natural water flow (5%).

**Figure 26:**
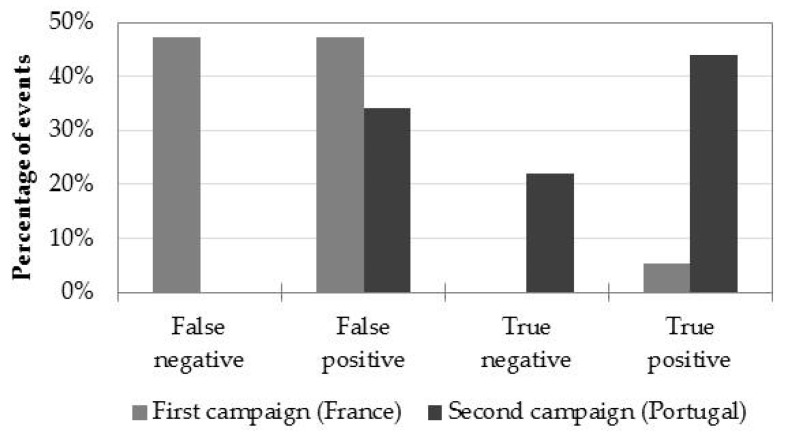
Distribution of the classified potential leak events (total number of events: 60).

**Figure 27:**
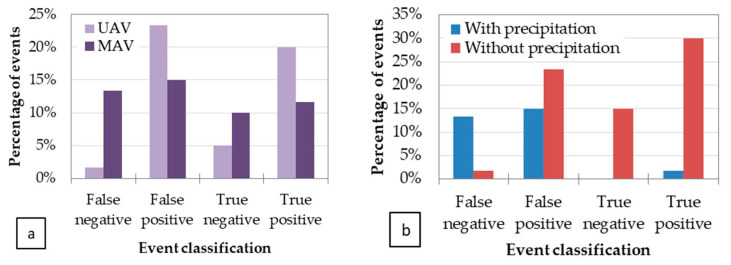
Relationship between event classification and (**a**) the platform used for the flights or (**b**) the occurrence of precipitation in the 10 days prior to the flights.

**Figure 28:**
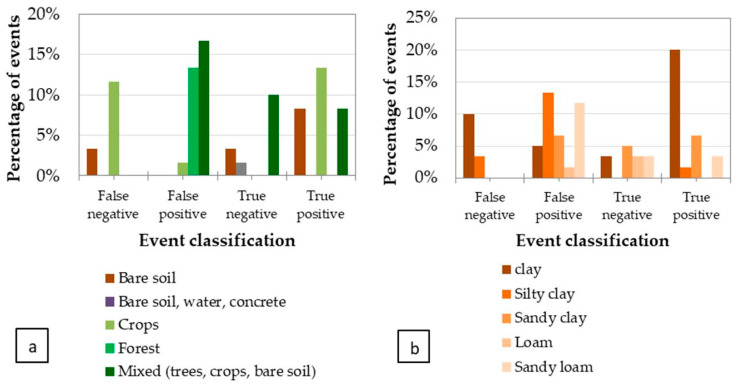
Relation between the event classification and (**a**) the type of vegetation or (**b**) the type of soil.

The performance matrix analysis was based on the classification of these 60 events as true positive/true negative/false positive/false negative events (see Figure 26), and on the influence of the parameters considered most relevant, namely, the technology used (UAV/MAV, see Figure 27a), the environmental conditions such as precipitation in the previous 10 days before the flight (Figure 27b), vegetation type (Figure 28a), and soil type (Figure 28b).

Overall, the T-VI multispectral system was able to successfully identify true events in approximately 50% of the occasions. There is a significant improvement in the performance of the method from the implementation (France) to the validation (Portugal) phases, with a 65% rate of correct detections (Figure 26). The implementation of the gyrostabilized platform in the MAV, and advances in the image processing workflow were the main responsible factors for that improvement.

Environmental conditions were also found to be important in the enhanced performance of the Portugal tests. Indeed, the analysis of the impact of the precipitation in the last ten days for both campaigns shows that 65% of the events were correctly detected under no precipitation conditions (Figure 27b).

Leaks in clay soils were found to be better detected than in sandy soils, although a mixed type (sandy clay soil) also performs well, as opposed to a silt–clay soils (Figure 28b). The analysis on the type of vegetation showed that the WADI service is not adequate for forested areas or their proximity, due to the smaller sensitivity of the thermal signal to the soil moisture and to the artefacts introduced by the shading caused by the trees, which complicates the interpretation of the images and is liable to generate false alarms. Agricultural zones characterized by bare soils, crops (at the early stage of development), and mixed areas are thus the most adequate places where WADI technology has the greatest capacity for success in detecting leaks (Figure 28a).

The type of flight technology used appears to perform equally well in leak detection (Figure 27a). Indeed, UAV and MAV platforms provide approximately the same fraction of true event classifications (true positives and true negatives). The main differences between the two choices are of course in terms of spatial coverage, spatial resolution, and exploitation cost, for which a compromise should be found. The MAV should thus be used in the first place for global analysis and in the pinpointing of the most relevant leak areas, letting UAVs operate for closer inspections of suspect areas that are difficult to access.

## 5. Conclusions

The Triangle/Trapezoid Method is a multispectral optical remote sensing method that is well known to be efficient for mapping evapotranspiration and soil moisture at the landscape (>1 km) and field scales (>100 m) from satellite images. Downscaling by using airplanes to get finer resolution information (e.g., better than 1 m) is still scarce and its application to water leak detection is indeed a pioneering approach. The application is innovative in this field since it is intended to provide raster information in a short time over water networks that span over large areas. At the same time, when changing from plane to drone, we can obtain images with a still better resolution (in the range of 5 cm) and concentrate on suspicious areas that do not allow easy access.

The principle of the method is based on the fact that leaks expose the soil to more intense evaporation because of its higher moisture, thus leading to a lower surface temperature. A temperature decrease is observed for vegetation as well, the reason is here an increase in leaf transpiration. The temperature is mapped from the air by a thermal infrared camera. However, the thermal response of vegetation is different from bare soil, thus making it difficult to obtain an unequivocal answer in terms of moisture content in the case of a pixel that is fully or partially covered with vegetation. The Triangle Method allows the empirical building of a scale between apparent temperature and soil moisture that varies continuously with the vegetation cover-fraction. The information on cover-fraction is obtained via a vegetation index, for example NDVI or OSAVI, which is computed from the relative difference in optical reflectance measured in the red and near-infrared spectral bands. These data are provided by a second camera providing images of the area in these two specific spectral bands. In the end, combining the three optical signals (red, near-infrared and thermal infrared) allows mapping of the so-called Water-Index. By construction, the constraints on the calibration of the signals are greatly relaxed. Any anomaly in the WI image which manifests as high values in the immediate vicinity of a network line is an indication of the existence of a potential leak.

A series of remote sensing campaigns with a motor-glider, an airplane, and a drone were performed from 2017 to 2019 over the SCP (France) and EDIA (Portugal) water transportation networks, first for defining the multispectral system aimed at detecting water leaks and then for validating it in operational conditions. In several sites, both artificial and natural leaks were present.

Hyperspectral and thermal cameras were first operated to get spectral images encompassing the VNIR, SWIR and TIR spectral bands. The best results were obtained by applying the Triangle Method by combining the thermal infrared image with a vegetation-index image built from two VNIR images. The contrasts obtained with the SWIR-based indexes and with the so-called Optical Trapezoid Method were lower. Spectrally optimized optical sensors were then operated onboard a small plane and a UAV to reach complementary performances regarding field scale and resolution. A preliminary multitemporal analysis showed that the identification of the leak signature could be optimized by carefully choosing the time of flight with respect to sun radiance history.

Possible false alarms come from high vegetation and variations in soil/vegetation optical properties that are not filtered out enough by the Triangle Method. Other sources of misinterpretation are vegetation shadows and infrastructures. Close interaction with the water utilities allows reducing the false alarm rate. Ancillary information can also help reject false alarms, for example, if the terrain is sloping and if the WI-anomaly extends from the nearby pipe in the uphill direction (beyond what simple water diffusion could induce). In order to ease the validation of the remote sensing results by on-site observation, the time between the flights and the in-situ checking should be short so that conditions are as equal as possible at both moments, at least with no rain event in between, which was not always the case during the present campaigns. This analysis should be reviewed and updated, whenever new flights can be made and new events are detected.

The performance of the leak detection method was satisfactory with a 50% event-detection accuracy. Several site characteristics contribute to success such as agricultural zones characterized by bare soils, crops (at the early stage of development) and mixed areas. The type of flight technology used appears to be equally well-suited for performing leak detections.

## Data Availability

Part of the collected data is available in the Public data repository Zenodo: https://zenodo.org/communities/h2020-wadiproject/?page=1&size=20 (accessed on 29 November 2021).

## References

[1] William S., Tamin P. (2013). Water Tech: A Guide to Investment, Innovation and Business Opportunities in the Water Sector.

[2] Office of the High Commissioner for Human Rights. https://www.ohchr.org/EN/NewsEvents/Pages/DisplayNews.aspx?NewsID=26595&LangID=E.

[3] European Commission (2012). A Blueprint to Safeguard Europe’s Water Resources.

[4] European Commission (2011). Water Is for Life: How the Water Framework Directive Helps Safeguard Europe’s Resources.

[5] Rizzo P. (2010). Water and wastewater pipe nondestructive evaluation and health monitoring: A review. Adv. Civ. Eng..

[6] Mazumder R.K., Salman A.M., Li Y., Yu X. (2018). Performance evaluation of water distribution systems and asset management. J. Infrastruct. Syst..

[7] Oh Y. (2004). Quantitative retrieval of soil moisture content and surface roughness from multipolarized radar observations of bare soil surfaces. IEEE Trans. Geosci. Remote Sens..

[8] Njoku E.G., Wilson W.J., Yueh S.H., Dinardo S.J., Li F.K., Jackson T.J., Bolten J. (2002). Observations of soil moisture using a passive and active low-frequency microwave airborne sensor during SGP99. IEEE Trans. Geosci. Remote Sens..

[9] Fabre S., Briottet X., Lesaignoux A. (2015). Estimation of soil moisture content from the spectral reflectance of bare soils in the 0.4–2.5 µm domain. Sensors.

[10] Zhang D., Zhou G. (2016). Estimation of soil moisture from optical and thermal remote sensing: A review. Sensors.

[11] Gerhards M., Schlerf M., Mallick K., Udelhoven T. (2019). Challenges and future perspectives of multi-/Hyperspectral thermal infrared remote sensing for crop water-stress detection: A review. Remote Sens..

[12] Myer V.I. (1975). Crops and soils. Manual of Remote Sensing.

[13] Schmugge T. (1978). Remote sensing of surface soil moisture. J. Appl. Meteorol..

[14] Jensen J.R. (2014). Remote Sensing of the Environment: An Earth Resource Perspective.

[15] Nellis M.D. (1982). Application of thermal infrared imagery to canal leakage detection. Remote Sens. Environ..

[16] Cundill S.L., Meijde M., Hack R.G.K. (2014). Investigation of remote sensing for potential use of dike inspection. IEEE J. Sel. Top. Appl. Earth Obs. Remote Sens..

[17] Tracey J.P., Walton P.A. (1989). Hydrologic investigations in canal and aqueduct systems using airborne thermal infrared linescanning. Proceedings of the IGARSS’89 12th Canadian Symposium on Remote Sensing Geoscience and Remote Sensing Symposium.

[18] Pickerill J.M., Malthus T.J. (1998). Leak detection from rural aqueducts using airborne remote sensing. Int. J. Remote Sens..

[19] McGowen I.J., Duff S.L., Smith I. (2001). Identifying channel seepage using pre-dawn thermal imagery. Proceedings of the IGARSS’01, IEEE 2001 International Geoscience and Remote Sensing Symposium.

[20] Huang Y., Fipps G., Maas S.J., Fletcher R.S. (2009). Airborne remote sensing for detection of irrigation canal leakage. Irrig. Drain..

[21] Thomson S.J., Ouellet-Plamondon C., DeFauw S.L., Huang Y., Fisher D.K., English P.J. (2012). Potential and challenges in use of thermal imaging for humid region irrigation system management. J. Agric. Sci..

[22] Charles N., Royet P., Meriaux P., Duval C. (2013). Etat des méthodes éprouvées et innovantes pour la surveillance des digues fluviales et maritimes. Colloque National, Digues Maritimes et Fluviales de Protection Contre les Submersions.

[23] Arshad M., Gomez R., Falconer A., Roper W., Summers M. (2014). A remote sensing technique detecting and identifying water activity sites along irrigation canals. Am. J. Environ. Eng. Sci..

[24] Moran M.S., Clarke T.R., Inoue Y., Vidal A. (1994). Estimating Crop water deficit using the relation between surface-air temperature and spectral vegetation index. Remote Sens. Environ..

[25] Carlson T.N., Gillies R.R., Schmugge T.G. (1995). An interpretation of methodologies for indirect measurement of soil water content. Agric. For. Meteorol..

[26] Sandholt I., Rasmussen K., Andersen J. (2002). A simple interpretation of the surface temperature-vegetation index space for assessment of surface moisture status. Remote Sens. Environ..

[27] Carlson T.N. (2007). An overview of the “triangle method” for estimating surface evapotranspiration and soil moisture from satellite imagery. Sensors.

[28] Petropoulos G.P., Carlson T.N., Wooster M.J., Islam S. (2009). A review of Ts/VI remote sensing based methods for the retrieval of land surface energy fluxes and soil surface moisture. Prog. Phys. Geogr..

[29] Krapez J.-C., Olioso A., Coudert B. Comparison of three methods based on the temperature-NDVI diagram for soil moisture characterization. Proceedings of the Remote Sensing for Agriculture, Ecosystems, and Hydrology XI.

[30] Chauhan N.S., Miller S., Ardanuy P. (2003). Spaceborne soil moisture estimation at high resolution: A microwave-optical/IR synergistic approach. Int. J. Remote Sens..

[31] Krapez J.-C., Olioso A. (2011). A combination of temperature, vegetation indexes and albedo, as obtained by airborne hyperspectral remote sensing, for the evaluation of soil moisture. J. Quant. Infrared Thermogr..

[32] Krapez J.-C., Chatelard C., Nouvel J.-F., Déliot P. (2012). Combined airborne thermography and visible-to-near infrared reflectance measurement for soil moisture mapping. Proceedings of the 11th International Conference on Quantitative InfraRed Thermography, QIRT 2012 Conference.

[33] Maltese A., Cammalleri C., Capodici F., Ciraolo G., Loggia G.L. Surface soil humidity retrieval using remote sensing techniques: A triangle method validation. Proceedings of the Remote Sensing for Agriculture, Ecosystems, and Hydrology XII.

[34] Maltese A., Capodici F., Ciraolo G., Loggia G.L. (2015). Soil water content assessment: Critical issues concerning the operational application of the triangle method. Sensors.

[35] Fan L., Xiao Q., Wen J., Liu Q., Tang Y., You D., Li X. (2015). Evaluation of the airborne CASI/TASI Ts-VI space method for estimating near-surface soil moisture. Remote Sens..

[36] Sanchez N., Piles M., Martínez-Fernández J., Vall-llossera M., Pipia L., Camps A., Herrero-Jiménez C.M. (2014). Hyperspectral optical, thermal, and microwave L-Band observations for soil moisture retrieval at very high spatial resolution. Photogram. Eng. Remote Sens..

[37] European Commission WADITECH, Innovative Airborne Water Leak Detection Surveillance Service. http://www.waditech.eu.

[38] Chatelard C., Krapez J.C., Barillot P., Déliot P., Frédéric Y.M., Pierro J., Serra G. Multispectral approach assessment for detection of losses in water transmission systems by airborne remote sensing. Proceedings of the HIC 2018, 13th International Conference on Hydroinformatics.

[39] Chatelard C., Muñoz J.S., Krapez J.C., Mazel C., Olichon V., Polo J.B., Serra G. (2019). Leak detection in water transmission systems by multispectral remote sensing with airplane and UAV. Proceedings of the IGARSS 2019-2019 IEEE International Geoscience and Remote Sensing Symposium.

[40] Krapez J.C., Muñoz J.S., Chatelard C., Mazel C., Olichon V., Polo J.B., Carvalho A. (2020). Assessment of the Triangle Method (T-VI) for Detection of Water Leaks from Airplane and UAV. Proceedings of the IGARSS 2020 International Geoscience and Remote Sensing Symposium.

[41] Brigot G., Colin-Koeniguer E., Plyer A., Janez F. (2016). Adaptation and evaluation of an optical flow method applied to co-registration of forest remote sensing images. IEEE J. Sel. Top. Appl. Earth Obs. Remote Sens..

[42] Peñuelas J., Pinol J., Ogaya R., Filella I. (1997). Estimation of plant water concentration by the reflectance water index WI (R900/R970). Int. J. Remote Sens..

[43] Zarco-Tejada P.J., Rueda C.A., Ustin S.L. (2003). Water content estimation in vegetation with MODIS reflectance data and model inversion methods. Remote Sens. Environ..

[44] Gao B.-C. (1996). NDWI—A normalized difference water index for remote sensing of vegetation liquid water from space. Remote Sens. Environ..

[45] Zarco-Tejada P.J., Ustin S.L. Modeling canopy water content for carbon estimates from MODIS data at land EOS validation sites. Proceedings of the IGARSS’01, International Geoscience and Remote Sensing Symposium.

[46] Chen D., Huang J., Jackson T.J. (2005). Vegetation water content estimation for corn and soybeans using spectral indices derived from MODIS near-and short-wave infrared bands. Remote Sens. Environ..

[47] Whalley W.R., Leeds-Harrison P.B., Bowman G.E. (1991). Estimation of soil moisture status using near infrared reflectance. Hydrol. Processes.

[48] Bryant R., Thomas D., Moran S., Holifield C., Goodrich D., Deefer T., Paige G., Williams D., Skirvin S. Evaluation of hyperspectral, infrared temperature and radar measurements for monitoring surface soil moisture. Proceedings of the First Interagency Conference on Research in the Watersheds.

[49] Haubrock S.N., Chabrillat S., Lemmnitz C., Kaufmann H. (2008). Surface soil moisture quantification models from reflectance data under field conditions. Int. J. Remote Sens..

[50] Nagler P.L., Daughtry C.S.T., Goward S.N. (2000). Plant litter and soil reflectance. Remote Sens. Environ..

[51] Daughtry C.S.T. (2001). Discriminating Crop Residues from Soil by Shortwave Infrared Reflectance. Agron. J..

[52] Kokaly R.F., Asner G.P., Ollinger S.V., Martin M.E., Wessman C.A. (2009). Characterizing canopy biochemistry from imaging spectroscopy and its application to ecosystem studies. Remote Sens. Environ..

[53] Merzlyak M.N., Gitelson A.A., Chivkunova O.B., Rakitin V.Y. (1999). Non-destructive optical detection of pigment changes during leaf senescence and fruit ripening. Physiol. Plant..

[54] Ren S., Chen X., An S. (2017). Assessing plant senescence reflectance index-retrieved vegetation phenology and its spatiotemporal response to climate change in the Inner Mongolian Grassland. Int. J. Biometeorol..

[55] Rondeaux G., Steven M., Baret F. (1996). Optimization of soil adjusted vegetation indices. Remote Sens. Environ..

[56] Sadeghi M., Babaeian E., Tuller M., Jones S.B. (2017). The optical trapezoid model: A novel approach to remote sensing of soil moisture applied to Sentinel-2 and Landsat-8 observations. Remote Sens. Environ..

[57] Jimenez Gonzalez D., Krapez J.-C., Mendes A., Oliveira A., Alves E., Sanchis Muñoz J., Barba Polo J., De Badts E. WADI D4.2—Software User’s Guide. https://www.waditech.eu/about/results/d4-2-software-user-s-guide.kl.

